# Expression, Purification, and Functional Exploration of an α-Galactosidase from *Akkermansia muciniphila*

**DOI:** 10.3390/foods14213790

**Published:** 2025-11-05

**Authors:** Teng Zuo, Ziqian Yin, Zhiguo Li, Zhihao Ren, Yaqiang Chen, Dahai Yu, Xuexun Fang

**Affiliations:** Key Laboratory for Molecular Enzymology and Engineering, Ministry of Education, School of Life Sciences, Jilin University, Changchun 130012, Chinayudahai@jlu.edu.cn (D.Y.)

**Keywords:** *Akkermansia muciniphila*, α-galactosidase, protein expression and purification, enzymatic reaction kinetics, molecular dynamics simulation

## Abstract

*Akkermansia muciniphila* (AKK) is a mucin-degrading gut symbiont with emerging probiotic potential. Among its carbohydrate-active enzymes, Amuc_0517, a glycoside hydrolase family 36 (GH36) protein, has been identified as a highly specific α-galactosidase. In this study, the Amuc_0517 gene was cloned into pET-28a(+), expressed in *Escherichia coli* BL21, and purified via Ni^2+^-NTA affinity chromatography. Bioinformatic analysis indicated the presence of a signal peptide and α-galactosidase domain. Enzyme assays confirmed its ability to cleave α-1,6-glycosidic bonds in pNPGal, with no detectable activity toward pNPGlu, and molecular dynamics simulations revealed stronger binding affinity and lower free energy with pNPGal, supporting its substrate specificity. Given that α-galactosidases are widely applied in the dairy industry to hydrolyze galactose-containing oligosaccharides in milk and whey, the biochemical features of Amuc_0517 suggest its potential as a novel biocatalyst for functional dairy processing and probiotic-enriched dairy product development.

## 1. Introduction

*Akkermansia muciniphila* (AKK) is a Gram-negative anaerobic bacterium residing in the mucus layer of the colon [1,2]. Since its discovery in 2004, AKK has been recognized for its multiple health-promoting functions, including enhancement of intestinal barrier integrity [3], modulation of immune responses [4], regulation of host metabolism [5,6], attenuation of inflammation, and participation in the gut–brain axis [7,8]. In recent years, research has expanded from live bacteria to heat-inactivated forms, cell components, and metabolites [9]. Among these, AKK-derived proteins have attracted growing attention due to their immunoregulatory, metabolic, and barrier-enhancing functions [9].

Several AKK proteins have been functionally characterized. For example, Amuc_1100, an outer membrane protein, activates Toll-like receptor 2 (TLR2), promotes anti-inflammatory cytokine secretion, and improves mucosal integrity [10,11], while P9, a mucinase, specifically cleaves mucin glycoproteins to release short-chain fatty acids that nourish epithelial cells [12]. These examples illustrate the functional diversity and potential therapeutic value of AKK-derived proteins.

AKK relies on a repertoire of glycoside hydrolases (GHs) to degrade mucin for colonization and nutrient acquisition [13]. Mucins are extensively O-glycosylated proteins, and cleavage of glycosidic bonds within their sugar chains is a critical step in their degradation [14]. AKK secretes galactosidases, fucosidases, and sialidases to sequentially hydrolyze glycosidic linkages in mucin oligosaccharides [15]. Among these, α-galactosidases are particularly noteworthy as they remove terminal galactosyl residues and have applications in both food processing [16] and biomedical fields [17].

Beyond human health, α-galactosidases have notable industrial applications, particularly in the dairy sector. They can hydrolyze α-galactosidic linkages in milk and whey, improving digestibility for lactose-intolerant consumers, reducing flatulence-causing oligosaccharides, and enabling the synthesis of galactooligosaccharides (GOSs) with prebiotic benefits [18]. Therefore, α-galactosidases with high substrate specificity and suitable pH/temperature profiles are of interest not only for gut mucin degradation but also for value-added dairy processing.

The present study focuses on the cloning, expression, purification, and functional characterization of Amuc_0517, a predicted GH36 α-galactosidase from *A. muciniphila*. The enzyme was heterologously expressed in *Escherichia coli* BL21 using the pET-28a(+) vector and purified via Ni^2+^-NTA affinity chromatography. Enzymatic activity was assessed using p-nitrophenyl-α-D-galactopyranoside (pNPGal). Molecular dynamics simulations further elucidated substrate binding preferences. These biochemical insights not only contribute to understanding AKK’s glycan utilization but also suggest the potential of Amuc_0517 as a novel biocatalyst for functional dairy applications, including the development of lactose-reduced or GOS-enriched dairy products.

## 2. Materials and Methods

### 2.1. Amino Acid Sequence Analysis

The amino acid sequence of Amuc_0517 was retrieved from the NCBI database and analyzed using the ProtParam (ExPASy server, Swiss Institute of Bioinformatics, Geneva, Switzerland; https://web.expasy.org/protparam/, accessed on 3 June 2025). tool on the ExPASy server (Swiss Institute of Bioinformatics, Geneva, Switzerland; https://www.expasy.org/, accessed on 3 June 2025) to predict its physicochemical properties (Table 1). Hydrophobicity and hydrophilicity were evaluated using ProtScale (ExPASy server, Swiss Institute of Bioinformatics, Geneva, Switzerland; https://web.expasy.org/protscale/, accessed on 3 June 2025), and the presence of a signal peptide was predicted using SignalP-6.0. Transmembrane domains were analyzed using TMHMM-2.0, and subcellular localization was predicted via ProtCompB (Softberry Inc., Mount Kisco, NY, USA; http://www.softberry.com/berry.phtml?topic=pcompb&group=programs&subgroup=proloc, accessed on 3 June 2025). Sequence alignment with known α-galactosidases was conducted to determine its family classification and conserved motifs. The tertiary structure of Amuc_0517 was predicted using AlphaFold 3, and structural modeling and annotation were performed in PyMOL3.1.6.1 (Schrödinger LLC, New York, NY, USA).

### 2.2. Vector Construction and Expression Optimization

The recombinant plasmid pET-28a(+)-Amuc_0517 (residues 22–675) was synthesized (Sangon Biotech, Shanghai, China) and transformed into *Escherichia coli* BL21(DE3) competent cells. Positive clones were screened on selective media, cultured, and verified by Sanger sequencing (Kumei Biotechnology, Changchun, China) to confirm correct sequence insertion without frameshift or point mutations. To optimize the expression of soluble recombinant Amuc_0517, three single-factor induction conditions were tested: induction temperature, IPTG concentration, and induction time.

Induction temperature: Cultures were grown to an OD_600_ = 0.6 and induced with 1.0 mM IPTG at 16 °C, 25 °C, or 37 °C for 16 h at 150 rpm.

IPTG concentration: IPTG was added at concentrations of 0.2, 0.4, 0.6, 0.8, 1.0, and 1.5 mM, with induction performed at 25 °C for 16 h.

Induction time: Induction was conducted for 4, 8, 12, or 16 h at 25 °C with 1.0 mM IPTG. Samples before and after induction, as well as supernatant and pellet fractions following cell lysis, were analyzed by SDS-PAGE.

### 2.3. Protein Purification, Identification, and Enzyme Activity Assay

Induced cells were harvested by centrifugation; every 1 g of cells was resuspended in 10 mL of Tris-HCl buffer (50 mM Tris, 300 mM NaCl) and lysed by ultrasonication. Purification of the target protein was performed using affinity chromatography, and the supernatant was incubated with a gravity flow column Ni^2+^-NTA Beads 6FF (Smart-Lifesciences, Changzhou, China) for 1 h on ice. The target protein was eluted sequentially with 10 mM, 100 mM, and 250 mM imidazole buffers at 4 °C and analyzed by SDS-PAGE. For Western blotting, 70–100 kDa protein bands were cut from the gel according to the protein size displayed by the protein markers (TRANS, Blue Plus II Protein Marker, 14–120 kDa) and transferred to PVDF membranes (Millipore, Burlington, MA, USA) using wet electrotransfer at 250 mA for 1.5 h. Membranes were blocked with 5% skim milk for 1 h at room temperature, incubated overnight at 4 °C with mouse anti-His monoclonal antibody (1:8000, Abmart, Shanghai, China), and then with HRP-conjugated goat anti-mouse IgG (1:5000, Biosharp, Hefei, China). Signals were visualized using a chemiluminescence imaging system (Tanon-5200, Tianneng Technology, Shanghai, China). Protein concentration was quantified using a BCA protein assay kit (Thermo Fisher, Waltham, MA, USA). A standard curve was generated with known BSA concentrations, and sample concentrations were calculated accordingly. Enzymatic activity was determined using pNPGal (Sigma-Aldrich, St. Louis, MO, USA) as substrate. Reactions (200 μL) containing 10 μL of enzyme solution (30 μg/mL), 50 μL of a pNPGal solution (1 mM), and 100 μL of pH 6.0 buffer were incubated at 37 °C for 10 min, terminated with 40 μL of 0.1 M Na_2_CO_3_, and measured at 405 nm in a microplate reader. Commercial α-galactosidase (Aladdin, Shanghai, China) was used as a positive control; its official specific activity is 2000 U/g. The release of p-nitrophenol (pNP) was quantified via a standard curve.

### 2.4. Optimization of Enzymatic Reaction Conditions

To determine the optimal catalytic conditions for Amuc_0517, the following parameters were evaluated using single-factor assays. (Unless otherwise specified, the protein loading capacity for each lane was 20 μg/mL):

Substrate specificity: pNPGal and pNPGlu were used as substrates under identical conditions to assess substrate preference.

Temperature: Reaction temperatures of 4 °C, 15 °C, 25 °C, 37 °C, 45 °C, 55 °C, and 65 °C were tested.

pH: Buffers of pH 2.0 to 8.0 were used to determine the optimal pH (increments of 1.0).

Enzyme concentration: Final enzyme concentrations of 0–35 μg/mL were tested.

Reaction time: Reactions were run for 0–80 min in 10-min intervals.

### 2.5. Molecular Dynamics (MD) Simulation

All MD simulations were performed using GROMACS 2022.3. The specific conditions and methods used in the experiment can be found in the Supplementary Materials under Section S1.1.

### 2.6. Enzyme Kinetics

The initial reaction velocity (V_0_) was determined using substrate concentrations ranging from 0 to 8 mM: 0, 0.1, 0.15, 0.2, 0.3, 0.4, 0.5, 1.0, 2.0, and 8.0 mM. Reactions were conducted under optimized conditions. The Michaelis–Menten parameters (K_m_ and V_max_) were calculated from Lineweaver–Burk plots using the equation of the1/V_0_ vs. 1/[S] curve.

### 2.7. Data Analysis

All experiments were performed in triplicate. Data are presented as the mean ± standard deviation (SD). Statistical analyses and curve fitting were conducted using Origin 2022 (OriginLab, Northampton, MA, USA).

## 3. Results

### 3.1. Amino Acid Sequence Analysis

**Figure 1:**
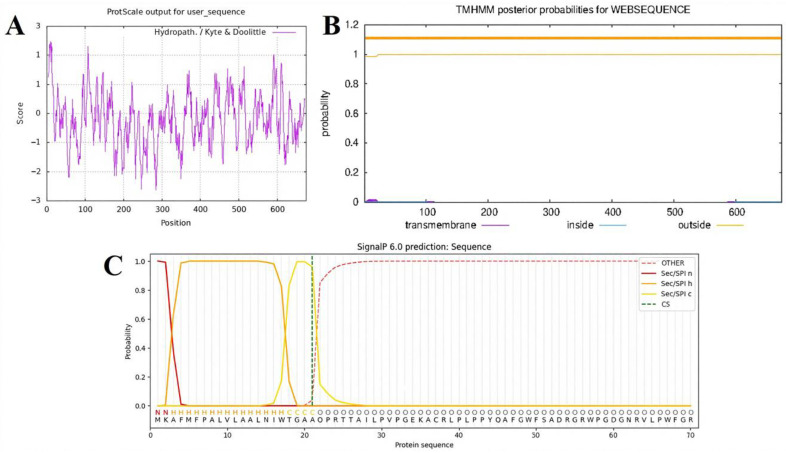
Analysis and prediction of basic properties of the Amuc_0517 protein. (**A**) Hydrophobicity profile prediction. (**B**) Transmembrane domain prediction (The upper thick orange band represents the posterior probability range for residues predicted to be located on the extracellular (outside) side of the membrane. The narrowness of the band indicates high confidence in the prediction, as the upper and lower probability limits nearly overlap, posterior probability ≈ 1.0). (**C**) Signal peptide sequence prediction.

Hydrophobic interactions play a key role in protein folding. Therefore, analysis of the hydropathic profile is an important first step in understanding protein structure and membrane association. The hydrophobicity of Amuc_0517 was assessed using ProtScale (ExPASy), where negative scores indicate hydrophilicity and positive scores indicate hydrophobicity. The results indicated that Amuc_0517 is a hydrophilic protein, with the most hydrophilic residue at position 284 (score = −2.633) and the most hydrophobic residue at position 10 (score = 2.467) (Figure 1A). TMHMM-2.0 analysis predicted that Amuc_0517 does not contain any transmembrane helices, suggesting that the entire protein is located outside the membrane (Figure 1B). SignalP-6.0 predicted a signal peptide between residues 1–21 with a 99.92% probability, and the cleavage site was predicted to be between residues 21 and 22 with 96.12% probability (Figure 1C). Based on these results, the N-terminal 21 amino acids were designated as the signal peptide. Subcellular localization analysis using ProtCompB further supported the classification of Amuc_0517 as a secreted protein. Based on the NNets, Pentamers, and Integral scoring systems, the highest scores were consistently assigned to the secreted compartment (Table 2).

Sequence alignment of Amuc_0517 with proteins in the NCBI database revealed five homologs with >40% sequence identity, all classified as Sip1-related α-galactosidases. These homologs included WP_045027438 [19], WP_250927797 and WP_168442090 [20], and WP_170162906 and WP_136078874 [21]. A phylogenetic tree constructed using these sequences showed that Amuc_0517 clustered closely with WP_250927797 and WP_168442090 within the same evolutionary clade. Notably, Amuc_0517 formed a unique leaf node, suggesting that it may represent a novel branch or a newly evolved member of this α-galactosidase family (Figure S1).

**Figure 2:**
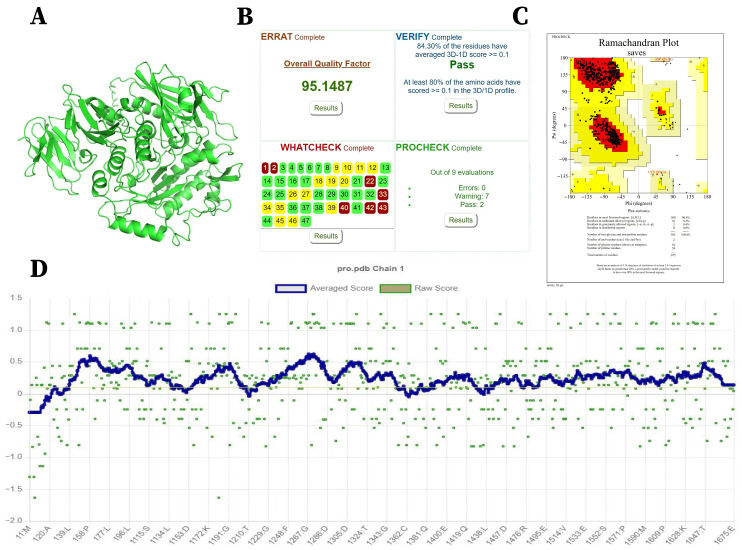
Three-dimensional Structure and Model Evaluation of Amuc_0517. (**A**) Predicted 3D structure of Amuc_0517 obtained using the AlphaFold 3 web server. (**B**) Protein structure evaluation results from UCLA-DOE LAB-SAVES v6.1. (**C**) Ramachandran plot. (**D**) Verify-3D score.

The tertiary structure of Amuc_0517 was predicted using AlphaFold 3. Given that the crystal structure of Amuc_0517 has not been resolved, this prediction serves as a valuable tool for investigating its catalytic mechanism. Since the crystal structure of Amuc_0517 has not yet been resolved, this limits structure-based investigations of its catalytic mechanism. Using the AlphaFold 3 web platform, we obtained a predicted 3D model of Amuc_0517 (Figure 2A). The protein sequence of Amuc_0517 used for AlphaFold 3 prediction is available in the Supplementary Materials under the Section S1.2. To assess the reliability of the model, we evaluated its quality using the third-party protein structure validation tool UCLA-DOE LAB-SAVES v6.1 [22].

The ERRAT analysis, which assesses protein model quality based on the Ramachandran plot, yielded a score of 95.1487, indicating that 95.1487% of the residues are located within the allowed regions—an exceptionally high score (Figure 2B). The Ramachandran plot analysis showed that 90.4% of residues fall within the most favored regions, 9.2% in additionally allowed regions, 0.4% in generously allowed regions, and 0% in disallowed regions (Figure 2C). Furthermore, as shown in Figure 2D, 84.3% of residues have a 3D-1D score ≥ 0.1, indicating that the prediction quality of the model is excellent.

Taken together, these evaluation metrics indicate that the predicted model exhibits very high overall structural quality.

**Figure 3:**
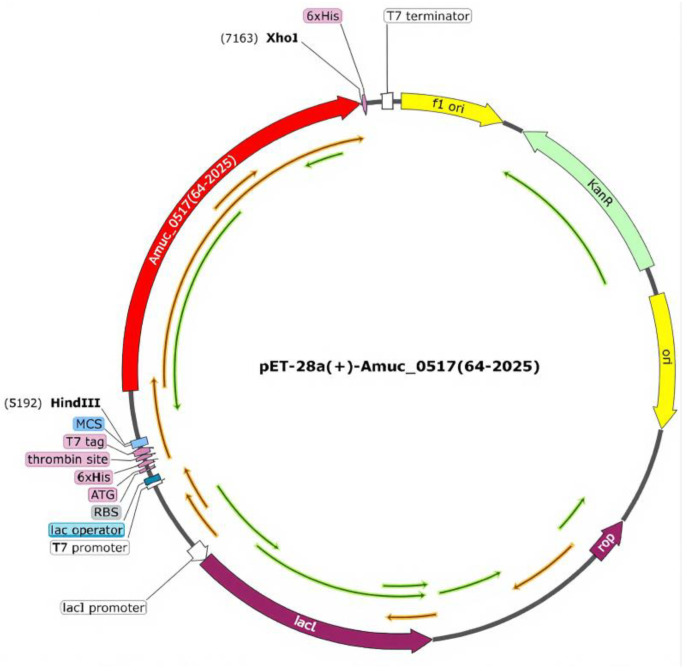
Plasmid map of pET-28a(+)-Amuc_0517 (64–2025).

Based on the above amino acid sequence, we constructed a gene expression vector (Figure 3) based on pET-28a(+) for the subsequent expression and purification of the target protein. The final recombinant protein contained not only the Amuc_0517 sequence but also additional elements at both termini, including 6×His tags, a thrombin cleavage site, a T7 tag, and a short peptide linker region. These small extensions resulted in a theoretical molecular weight of 77.756 kDa (approximately 78 kDa), which is slightly higher than that of the native Amuc_0517 (74.233 kDa). The complete amino acid sequence of the recombinant protein is provided in the Supplementary Materials.

### 3.2. Optimization of Expression Conditions

**Figure 4:**
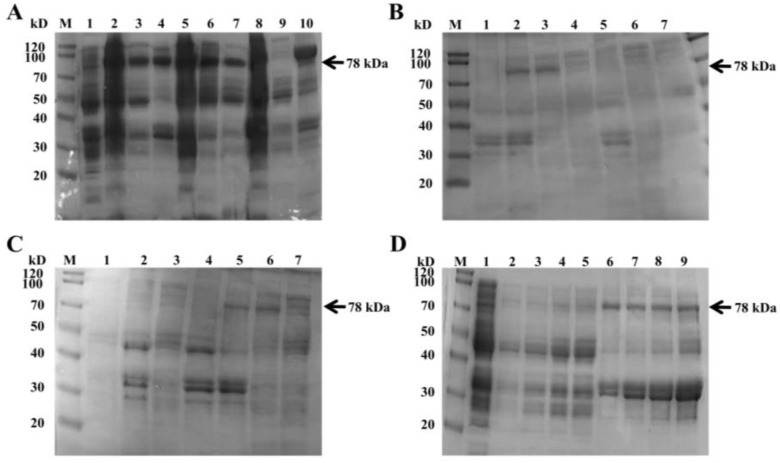
Optimization of recombinant protein expression by SDS-PAGE. (**A**) Induction temperature optimization. Lane 1: uninduced cells; Lane 2: induced at 16 °C; Lane 3: 16 °C supernatant; Lane 4: 16 °C pellet; Lane 5: 25 °C induced cells; Lane 6: 25 °C supernatant; Lane 7: 25 °C pellet; Lane 8: 37 °C induced cells; Lane 9: 37 °C supernatant; Lane 10: 37 °C pellet. (**B**) SDS-PAGE of supernatants at different IPTG concentrations. Lane 1: 1.5 mM; Lane 2: 1.0 mM; Lane 3: 0.8 mM; Lane 4: 0.6 mM; Lane 5: 0.4 mM; Lane 6: 0.2 mM; Lane 7: uninduced cells; (**C**) SDS-PAGE of pellets at different IPTG concentrations. Lane 1: uninduced cells; Lane 2: 0.2 mM; Lane 3: 0.4 mM; Lane 4: 0.6 mM; Lane 5: 0.8 mM; Lane 6: 1.0 mM; Lane 7: 1.5 mM. (**D**) Time-course of induction. Lane 1: uninduced cells; Lane 2–5: pellets at 4, 8, 12, and 16 h; Lanes 6–9: supernatants at 4, 8, 12, and 16 h (For all experiments, the protein content added to each lane is 20 μg).

SDS-PAGE analysis showed that the protein was expressed at all tested temperatures (16 °C, 25 °C, 37 °C), but expression levels varied (Figure 4A). At 25 °C, the highest amount of soluble protein was detected. At 37 °C, although the protein was expressed, most of it was found in the insoluble pellet as inclusion bodies. Therefore, temperature significantly influenced the soluble expression of Amuc_0517 (Figure 4A) and 25 °C was selected as the optimal induction temperature. Varying IPTG concentrations also affected protein yield (Figure 4B,C). All tested IPTG concentrations (0.2 to 1.5 mM) induced expression, and 0.8 mM IPTG gave the highest level of soluble protein. Higher IPTG concentrations resulted in reduced protein yield, likely due to cellular stress or inclusion body formation caused by rapid overexpression [23]. Under the condition of 25 °C, a certain amount of protein expression can be obtained by inducing for 4 h. Although the total protein content increases with the extension of induction time, the level of target protein band remained stable. Therefore, 4 h was chosen as the optimal induction time (Figure 4D).

### 3.3. Purification, Identification, and Enzymatic Assay of Amuc_0517

**Figure 5:**
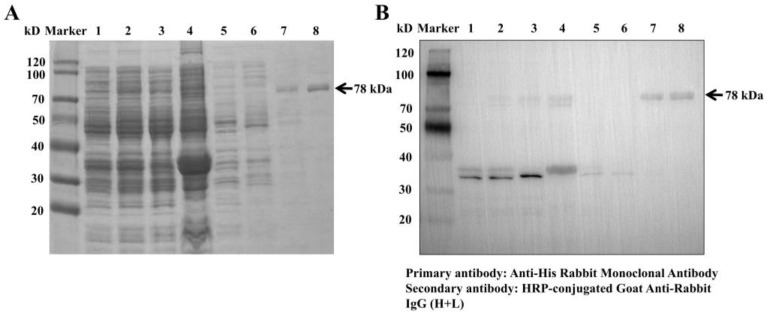
(**A**) SDS-PAGE of recombinant Amuc_0517 purifications. (**B**) Western blot analysis of recombinant Amuc_0517 purifications using anti-His antibody. Lane 1: uninduced cells; Lane 2: IPTG-induced cells; Lane 3: bacterial lysate supernatant; Lane 4: bacterial debris pellet; Lane 5: cell lysate flowthrough; Lanes 6–8: 10, 100, and 250 mM imidazole elutions.

Figure 5A shows the SDS-PAGE result followed by Ni^2+^-NTA column purification. Non-specific proteins were eluted with 10 and 100 mM imidazole, while the target protein was most enriched in the 250 mM fraction. The eluate was dialyzed in Tris-HCl buffer for 24 h to remove imidazole and then concentrated using a 30 kDa ultrafiltration tube.

Western blotting confirmed that the purified protein specifically bound to anti-His antibodies, indicating successful expression and purification, and the purity of the product reached over 90% (Figure 5B). BCA assay yielded a standard curve of y = 0.0008x + 0.0861 (R^2^ = 0.9911), and the final protein concentration was 1400 μg/mL (6.25 mg of target protein was obtained per liter of culture medium). For enzyme activity detection, we used pNPGal, a specific substrate of glycosidase, to detect enzyme activity, which was measured by detecting the content of pNP, the product of the enzymatic reaction. Based on the standard curve for pNP (y = 0.0117x + 0.0045, R^2^ = 0.9998), the specific activity of Amuc_0517 was calculated to be 170 U/mg.

### 3.4. Optimization of Enzymatic Reaction Conditions

**Figure 6:**
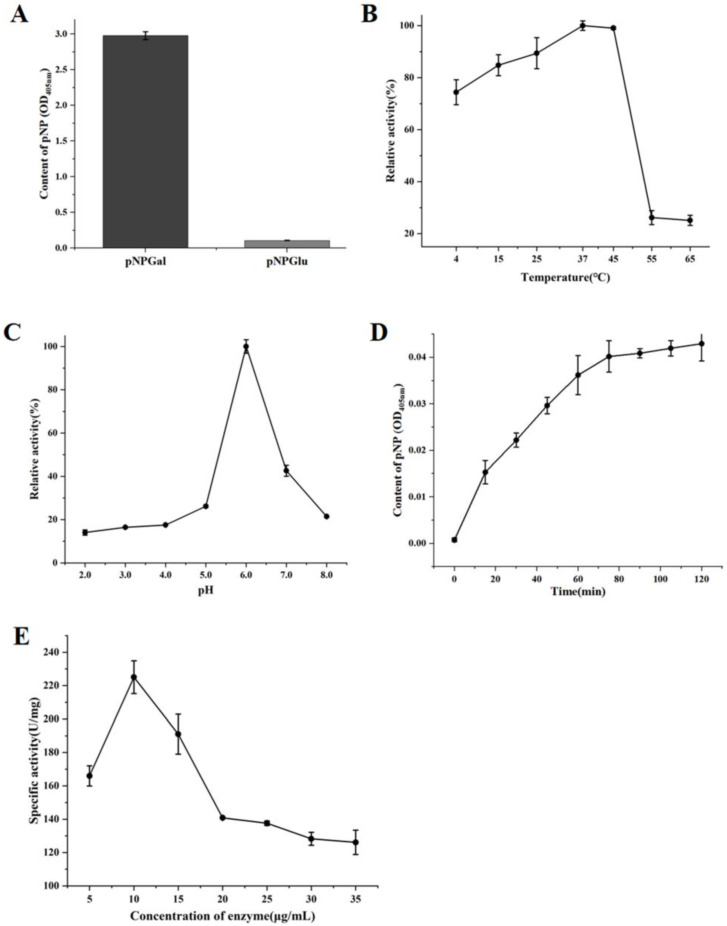
Enzymatic Reaction Characteristics of Amuc_0517 α-Galactosidase. (**A**) Substrate specificity of Amuc_0517 toward pNPGal and pNPGlu. (**B**) Optimal temperature screening from 4 °C to 65 °C. (**C**) Optimal pH screening from 2.0 to 8.0. (**D**) Optimal reaction time from 0 to 120 min. (**E**) Optimal enzyme concentration from 5 to 35 μg/mL.

Substrate specificity was evaluated using pNPGal and pNPGlu (Figure 6A). Amuc_0517 hydrolyzed pNPGal but showed no activity toward pNPGlu, confirming its α-galactosidase activity. Temperature optimization showed the highest enzymatic activity at 37 °C (Figure 6B). pH optimization revealed a peak activity at pH 6.0 (Figure 6C). Time-course analysis demonstrated that product formation plateaued after 80 min, but the initial reaction rate was highest between 0–10 min. Therefore, 10 min was chosen as the optimal reaction time (Figure 6D). The optimal enzyme concentration was determined to be 10 μg/mL, with a specific activity reaching 225 U/mg (Figure 6E). Higher concentrations led to substrate competition and reduced catalytic efficiency, while lower concentrations limited reaction speed.

### 3.5. Molecular Dynamics Simulation of Amuc_0517

**Figure 7:**
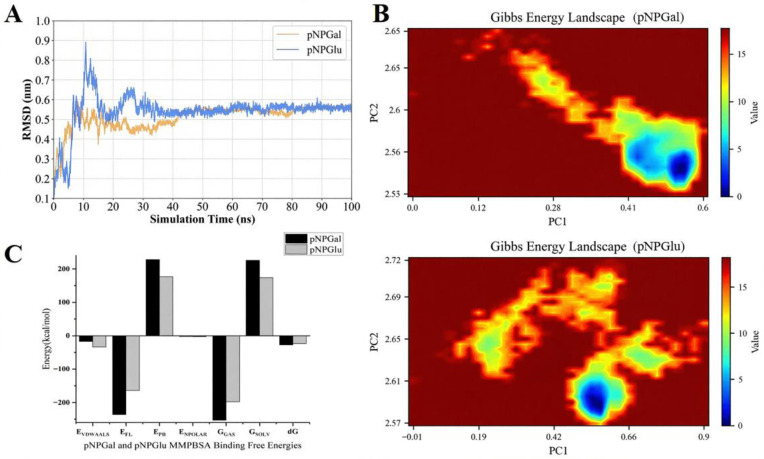
Structural and energetic basis for the substrate preference of Amuc_0517. (**A**) RMSD of the protein Cα atoms relative to the initial structure over 100 ns MD trajectories. The Amuc_0517–pNPGal complex (yellow) rapidly converged and remained below 0.4 nm, whereas the pNPGlu complex (blue) exhibited persistent drift up to 0.6 nm. (**B**) Gibbs free-energy landscapes projected onto PC1 and Rg. The pNPGal system (**above**) populates a single, narrow basin centered at PC1 ≈ 0.2 and Rg ≈ 2.55 nm (ΔG = 0 kJ mol^−1^); the pNPGlu system (**below**) spans multiple dispersed basins with higher Rg (≥ 2.65 nm) and energy barriers ≥ 10 kJ mol^−1^. (**C**) MM/PBSA decomposition of the binding free energies. The total ΔG_bind was −200 ± 5 kJ mol^−1^ for pNPGal and −100 ± 6 kJ mol^−1^ for pNPGlu; the more favorable interaction with pNPGal is dominated by electrostatics interaction energy (EEL) and van der Waals (EVDWAALS) contributions. Error bars represent the standard error of the mean over five 20-ns blocks.

As shown in Figure 7A, the Root-Mean-Square Elevation (RMSD) of the Amuc_0517–pNPGal complex converged within 20 ns and remained below 0.4 nm, whereas the Amuc_0517–pNPGlu complex exhibited continuous drift, reaching nearly 0.6 nm, indicating improved structural stability upon binding to the galactose substrate. The 2D free energy landscape based on principal component analysis (PCA) (Figure 7B) revealed that the pNPGal-bound state resided in a single, low-energy basin around Rg ≈ 2.55 nm (ΔG = 0 kJ/mol), whereas the pNPGlu-bound state occupied multiple high-energy regions (ΔG ≥ 10 kJ/mol), suggesting less stable binding conformations. MM/PBSA calculations (Figure 7C) indicated that the binding free energy for pNPGal was approximately −200 kJ/mol, nearly twice that of pNPGlu. The main energy difference was attributed to stronger van der Waals and electrostatic interactions. Further analyses of per-residue flexibility, radius of gyration (Rg), solvent-accessible surface area (SASA), and supplementary free energy landscapes are provided in Figure S2.

### 3.6. Enzyme Kinetic Analysis

**Figure 8:**
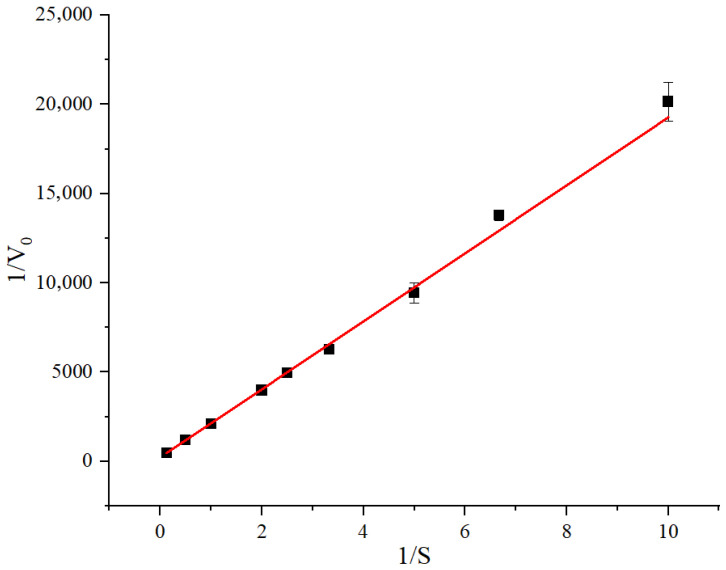
Determination of the Michaelis-Menten constant of Amuc_0517 with pNPGal as substrate. Data were calculated by nonlinear regression fitting with the enzyme Kinetics Module of Origin 2022 (OriginLab, USA). Means ± SD of 3 independent measurements are presented.

The enzyme kinetics of Amuc_0517 were evaluated to characterize its catalytic efficiency (Figure 8). A Lineweaver–Burk plot showed a strong linear relationship between 1/V_0_ and 1/[S], with the regression equation y = 1903.946x + 234.268 (R^2^ = 0.9981). Based on this, the V_max_ of Amuc_0517 was calculated to be 6.02 U/mg, and the K_m_ was determined as 8.2 mM. These values are comparable to those reported for other α-galactosidases in the literature [24]. A comparison of K_m_ and V_max_ values for β-galactosidase and β-glucosidase from different sources is presented in Table 3.

## 4. Discussion

In this study, we investigated the protein Amuc_0517 derived from AKK, systematically analyzing its expression, purification, enzymatic properties, substrate specificity, and catalytic mechanism. Additionally, we employed MD simulations to uncover the molecular basis of its substrate selectivity, providing a theoretical foundation for understanding AKK’s metabolic strategies in mucin degradation within the host gut.

Bioinformatics analysis first confirmed that Amuc_0517 contains a signal peptide sequence and is predicted to be localized extracellularly, indicating it is a secreted glycosidase. Previous studies have shown that AKK heavily relies on exogenous mucin as its carbon source, and its genome encodes numerous glycosidases, phosphotransferases, and oligosaccharide transport systems with signal peptides. These secreted enzymes work in concert to degrade and utilize mucin [30,31]. In our study, Amuc_0517 was successfully expressed in *Escherichia coli* BL21 and purified using a Ni^2+^-NTA column to obtain high-purity protein. Enzymatic assays confirmed that it exhibits α-galactosidase activity, capable of hydrolyzing the substrate pNPGal to release p-NP; its reaction activity was quantitatively monitored as changes in absorbance at 405 nm. Substrate specificity analysis revealed that Amuc_0517 could hydrolyze pNPGal, which contains a galactose moiety, but not the structurally similar pNPGlu that contains a glucose moiety, indicating a strong selectivity in recognizing the stereochemical configuration of the hydroxyl group at the C4 position [32]. Literature reports suggest that the substrate recognition ability of glycosidases often depends on the conformational adaptability of key residues in the active pocket and their selective exclusion of incompatible stereoisomers [33,34]. To elucidate the molecular basis of this selectivity, we performed MD simulations, free energy landscape (FEL) analysis, and binding free energy calculations using MM/PBSA. MD simulations showed that the Amuc_0517–pNPGal complex maintained conformational stability throughout the 100 ns simulation, with the RMSD fluctuating minimally around an average of 1.8 Å, indicating a stable protein structure and tightly bound substrate. In contrast, the Amuc_0517–pNPGlu complex exhibited greater RMSD fluctuations (~2.9 Å), especially in the later stages of the simulation, suggesting unstable binding conformations. This discrepancy is likely due to the altered orientation of the hydroxyl group at C4 in pNPGlu, which disrupts the hydrogen bond network in the active site, leading to looser binding or partial dissociation. FEL (free energy landscape) analysis further revealed significant differences in energy landscapes between the two complexes. The Amuc_0517–pNPGal complex displayed a single, deep, and narrow energy basin in the 2D energy map, indicating a concentrated and stable conformational state with a favorable energy profile. In contrast, the Amuc_0517–pNPGlu complex showed multiple shallow and dispersed energy basins, reflecting greater conformational diversity and lack of a clearly stable state. These differences in energy landscapes highlight the distinct structural adaptability of the enzyme active site when binding different substrates. The MM/PBSA binding free energy analysis supported these observations, with Amuc_0517 showing a significantly lower binding free energy to pNPGal than to pNPGlu (−34.6 kcal/mol vs. −18.2 kcal/mol), indicating a thermodynamically more favorable and stable complex formation with the former. Decomposition analysis further showed that key residues such as Trp312, Asp147, and Glu182 formed stable hydrogen bonds or hydrophobic interactions with pNPGal, resulting in a tightly embedded substrate conformation. In contrast, these interactions were substantially reduced or absent in the pNPGlu complex, leading to weaker binding. This aligns well with previous reports on α-galactosidases that recognize galactose features via aromatic residues and anionic groups [35,36].

Kinetic analysis revealed that Amuc_0517 exhibits maximum catalytic activity at 37 °C and pH 6.0, with a K_m_ of 8.2 mM and a V_max_ of 0.0043 μM/min. These optimal conditions are not only consistent with the mucosal environment of the gut but also align with conditions commonly encountered in dairy processing, particularly in fermented milk and yogurt production, where incubation temperatures range from 30–45 °C and the pH gradually drops to near 6.0 in early fermentation stages. Many α-galactosidases applied in the dairy industry are required to function effectively under moderate temperatures and slightly acidic pH to hydrolyze α-galactosidic linkages in milk and whey, thus reducing anti-nutritional oligosaccharides such as raffinose and stachyose, improving product digestibility, and facilitating the synthesis of GOSs with prebiotic functions [37].

Given its substrate specificity, Amuc_0517 could be explored as a biocatalyst for the selective removal of α-galactosyl residues from galactose-containing oligosaccharides in dairy matrices. Such activity could improve the nutritional and functional quality of milk-based products by (i) lowering flatulence-causing compounds for sensitive consumers, (ii) generating GOSs that selectively stimulate beneficial gut bacteria such as *Bifidobacterium* and *Lactobacillus*, and (iii) enhancing the overall prebiotic value of fermented dairy products [38].

Additionally, the enzyme’s predicted extracellular localization suggests that it could be secreted in recombinant dairy fermentation systems, potentially enabling in situ oligosaccharide conversion during yogurt or kefir production. Compared with other glycosidases discovered from bacteria, the optimum reaction temperature of Amuc_0517 is relatively narrow, which may limit its application in high-heat processes such as pasteurization [25]; however, this could be advantageous in post-pasteurization fermentation steps where mild processing preserves enzyme activity. Protein engineering strategies, including site-directed mutagenesis and directed evolution, could be employed to enhance its thermal stability or broaden its pH tolerance, making it more versatile for diverse dairy-processing conditions. Moreover, immobilization on food-grade carriers could facilitate enzyme recovery and reuse, reducing processing costs while maintaining activity in continuous lactose or oligosaccharide hydrolysis systems.

## 5. Conclusions

In conclusion, Amuc_0517 is a secreted α-galactosidase from AKK that exhibits precise regulation in substrate recognition, conformational adaptation, and thermodynamic stability, shedding light on the molecular adaptation strategies employed by AKK in complex glycan degradation. Beyond its biological role in mucin utilization, its biochemical features and optimal activity profile suggest potential applications in synthetic biology, probiotic enhancement, and functional dairy processing—particularly in the development of lactose-reduced, GOS-enriched, and gut-friendly dairy products.

The limitation of this study is that it did not investigate the degradation activity of the enzyme on various natural carbohydrate substrates, and only confirmed its ability to degrade galactosides through substrate synthesis. In addition, the stability range of the enzyme is relatively narrow, and its stability can be enhanced through further protein engineering modifications, expanding its application prospects in fields such as food processing.

## Figures and Tables

**Table 1:** Biochemical properties of Amuc_0517.

Biochemical Parameter	Value(s) of Biochemical Parameter
Amino acid residues	675
Molecular formula	C3362H5216N914O939S24
Theoretical molecular weight (Da)	74233.40 Da
Theoretical isoelectric point (pI)	8.94
Most abundant residues	Leu (10.8%), Ala (10.7%), Pro (8.6%)
Instability index	43.45
Aliphatic index	83.88
Grand average of hydropathicity	−0.216

**Table 2:** Subcellular localization prediction of Amuc_0517.

Location Weight	NNet	Pentamer Score	Integral
Cytoplasmic	0.00	0.00	0.00
Membrane	0.07	0.13	0.41
Secreted	0.96	1.88	6.08
Periplasmic	1.97	1.09	3.51

NNets: scores assigned by neural networks. Pentamers: scores based on comparisons of pentamer distributions calculated for QUERY and DB sequences. Integrals are final scores that combine all the other scores using a final neural net. The scores are renormalized to make a sum of scores for all localizations equal to 3 (NNets), 5 (PotLocDB, Pentamers) or 10 (LocDB, Integral).

**Table 3:** Comparison of K_m_ and V_max_ Values for β-Galactosidase and β-Glucosidase Enzymes from Various Sources.

Sources	Substrate(s)	K_m_ (mM)	V_max_ (U/mg)	Reference
*Akkermansia muciniphila*	pNPGal	8.20	6.02	This study
*Bacillus velezensis*	lactose	18.64	60.78	[25]
	oNPGal	1.95	89.62	
*Anoxybacillus* sp. DT3-1	pNPGlu	0.22	923.70	[26]
*Thermoanaerobacterium aotearoense* P8G3#4	glucose	0.66	180.60	[27]
*Nasutitermes takasagoensis*	glucose	0.67	8.00	[28]
*Rhizomucor miehei* NRRL 5282	glucose	0.12	468.20	[29]

## Data Availability

The original contributions presented in this study are included in the article/Supplementary Materials. Further inquiries can be directed to the corresponding author.

## Supplementary Materials

The following supporting information can be downloaded at: https://www.mdpi.com/article/10.3390/foods14213790/s1, Section S1: Supplementary method; Section S1.1: Molecular dynamics simulation; Section S1.2: Protein sequence for AlphaFold 3 prediction; Section S1.3: The amino acid sequence of the final recombinant protein; Section S2: Supplementary Figs; Figure S1: Phylogenetic tree analysis of AKK derived α-galactosidase Amuc_0517. Where “leaf node” represents the protein database number of each enzyme (except Amuc_0517); Figure S2: (A) Per-residue RMSF of Amuc_0517 during 100 ns MD simulations. The catalytic loop (residues 220–240) exhibits markedly lower flexibility in the pNPGal complex (yellow) than in the pNPGlu complex (blue). (B) Time evolution of Rg showing that the enzyme remains more compact (≈2.1 nm) with pNPGal (yellow) and more expanded (≈2.6 nm) with pNPGlu (blue). (C) SASA trajectories. The pNPGal complex maintains a smaller and more stable SASA (~245 Å²), whereas the pNPGlu complex exhibits higher and fluctuating values (~275 Å²). (D) Two-dimensional Gibbs free-energy landscapes (ΔG) as a function of RMSD and Rg for Amuc_0517 in complex with pNPGal (left) and pNPGlu (right). The galactoside-bound enzyme populates a single, narrow basin (ΔG = 0 kJ mol^−1^; RMSD ≈ 0.1 nm, Rg ≈ 2.56 nm), whereas the glucoside-bound enzyme exhibits multiple dispersed basins with higher energy barriers (ΔG ≥ 10 kJ mol^−1^) and increased Rg (≥2.64 nm), indicating greater conformational heterogeneity.
