# Supplementary material for: Expression, Purification, and Functional Exploration of an α-Galactosidase from Akkermansia muciniphila
Source: Foods. 2025 Nov 5;14(21):3790. doi: 10.3390/foods14213790 (PMC12610585; doi:10.3390/foods14213790)
Supplement: Supplementary file 1 [file foods-14-03790-s001.zip › foods-3886911-supplementary.pdf]

Expression, purification, and functional exploration of an α-galactosidase from *Akkermansia muciniphila*

Teng Zuo, Ziqian Yin, Zhiguo Li, Zhihao Ren, Yaqiang Chen, Dahai Yu and Xuexun Fang *

Key Laboratory for Molecular Enzymology and Engineering, Ministry of Education, School of Life Sciences, Jilin University, Changchun 130012, China

* Correspondence: Corresponding author. E-mail address: fangxx@jlu.edu.cn (X. Fang)

S1. Supplementary method

S1.1. Molecular dynamics simulation

Specific conditions and methods used in molecular dynamics simulation experiments. The structure of Amuc_0517 was modeled using AlphaFold 3 (The protein sequence used for prediction can be found in the supplementary materials) and refined via MODELLER 10.2. Substrates pNPGal and pNPGlu were docked into the active site using AutoDock Vina, and the highest-scoring binding pose was used as the initial structure. Complexes were solvated in a 10 Å dodecahedral TIP3P water box, neutralized with Na+/Cl- ions to 0.15 M. The Amber99SB-ILDN force field was applied to the protein, and GLYCAM_06j-1 parameters were used for the glycoside ligands. Energy minimization was per-formed by steepest descent until maximum force was below 1000 kJ/mol·nm. Two equilibration steps were conducted:

(1) 1 ns under NVT conditions at 310 K using the V-rescale thermostat;

(2) 1 ns under NPT conditions at 1 bar using the Parrinello–Rahman barostat.

After positional restraints were removed, 100 ns production runs were performed with 2 fs time steps. LINCS algorithm was applied to constrain hydrogen bonds, and long-range electrostatics were calculated using the particle mesh Ewald method (grid spacing 1.2 Å). Simulation data were saved every 10 ps. Root Mean Square Deviation (RMSD), Root Mean Square Fluctuation (RMSF), radius of gyration (Rg), and solvent-accessible surface area (SASA) were analyzed. A 2D free energy landscape was generated using the first principal component (PC1) and Rg. The landscape was

normalized to the global energy minimum using gmx covar and gmx anaeig with a grid spacing of 0.5 kJ/mol. MM/PBSA calculations were performed using the last 80 ns of the trajectory (8000 frames). Solvent dielectric constant was set to 80, solute to 4. Energy contributions included van der Waals (EvdW), electrostatic (EEL), polar solvation (EPB), and non-polar solvation (ENPOLAR). Errors were calculated from the standard error of five 20-ns blocks.

S1.2. Protein sequence for AlphaFold 3 prediction

MKAFMFPALVLAALNIWTGAAQPRTTAILPVPGEKACRLPLPPYQAFCWFSAD

RGRWPGDGNRVLPWFGRTAPGNLLDSKPNPSTAKPGDHAMFALFHLKDGNF

MAVLPVAAPDSLAWLKLERDGTFLVEAGSLGTSPAKPQAVLAVTATDKDIYRA

CSAVWDKALSLPFIKGRTLPREKKIYPEPFKYLGWCSWEQYKKNISSKLLEET

ARKLEASPVPVRWMLVDDGFQTQERLQLVSFQPRQDQFPRGWQPLMKHKSP

KLKWMGLWHCYYGLWNGIHPRHRLDDETARGLVRTAKGKILPGDGSGGAG

AFYTPFLQSVKDTGFDFVKIDVQAEYLKHADGLDNPVRHNTKCSEALEQACL

KTGLSLVNCMAQGTVNIQNTRYSAVTRCSIDYKLGDEAMAKSHILQSYANTL

WLGQTVWPDHDMFHSTDPACARLMAVSKAVSGGPVYLSDPADKLNPENIMP

LVWSDGLLLRPLAPAVPLPDSVFPDALNENRLYRVIAPLPGQSAAVVVYNLKH

PSPAEPVRGKISLEDYKNAAALLNGNAAEAYASLPAEGIAAYSAEGGRALTPA

QPDLDVELTGFKDRLFIMAPIVQGWAVIGRRDKFLSPCALVSAPGYRENGLRF

RVKESGPVVIWRGKGPVKAGNTPVRNLGNGFYELQFPVSDHPLDITVTAE

S1.3. The amino acid sequence of the final recombinant protein

MGSSHHHHHHSSGLVPRGSHMASMTGGQQMGRGSEFELRRQACQPRTTAILP

VPGEKACRLPLPPYQAFCWFSADRGRWPGDGNRVLPWFGRTAPGNLLDSKPN

PSTAKPGDHAMFALFHLKDGNFMAVLPVAAPDSLAWLKLERDGTFLVEAGSL

GTSPAKPQAVLAVTATDKDIYRACSAVWDKALSLPFIKGRTLPREKKIYPEPFK

YLGWCSWEQYKKNISSKLLEETARKLEASPVPVRWMLVDDGFQTQERLQLVS

FQPRQDQFPRGWQPLMKHKSPKLKWMGLWHCYYGLWNGIHPRHRLDDETA

RGLVRTAKGKILPGDGSGGAGAFYTPFLQSVKDTGFDFVKIDVQAEYLKHAD

GLDNPVRHNTKCSEALEQACLKTGLSLVNCMAQGTVNIQNTRYSAVTRCSID

YKLGDEAMAKSHILQSYANTLWLGQTVWPDHDMFHSTDPACARLMAVSKAV

SGGPVYLSDPADKLNPENIMPLVWSDGLLLRPLAPAVPLPDSVFPDALNENRL

YRVIAPLPGQSAAVVVYNLKHPSPAEPVRGKISLEDYKNAAALLNGNAAEAY

ASLPAEGIAAYSAEGGRALTPAQPDLDVELTGFKDRLFIMAPIVQGWAVIGRRD

KFLSPCALVSAPGYRENGLRFRVKESGPVVIWRGKGPVKAGNTPVRNLGNGF

YELQFPVSDHPLDITVTAELEHHHHHH

S2. Supplementary Figs

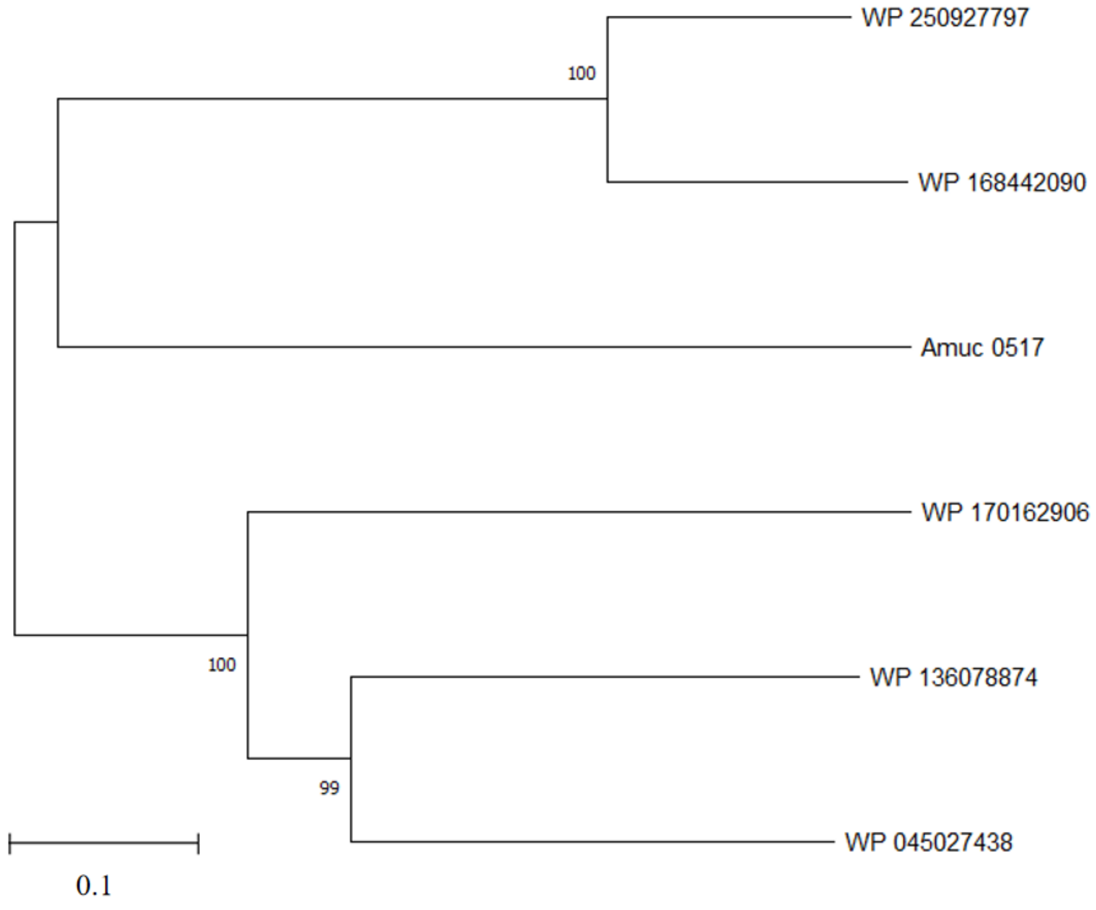

Figure S1. Phylogenetic tree analysis of AKK derived α-galactosidase Amuc_0517. Where "leaf node" represents the protein database number of each enzyme (except Amuc_0517).

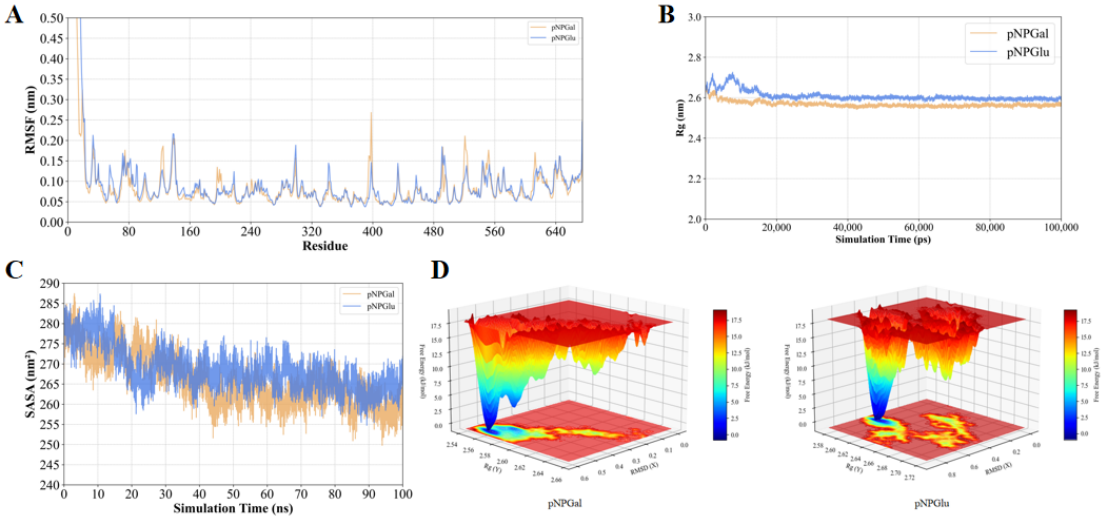

Figure S2. (A) Per-residue RMSF of Amuc_0517 during 100 ns MD simulations. The catalytic loop (residues 220–240) exhibits markedly lower flexibility in the pNPGal complex (yellow) than in the pNPGlu complex (blue). (B) Time evolution of Rg showing that the enzyme remains more compact (≈ 2.1 nm) with pNPGal (yellow) and more expanded (≈ 2.6 nm) with pNPGlu (blue). (C) SASA trajectories. The pNPGal complex maintains a smaller and more stable SASA (~245 Å^2), whereas the pNPGlu complex exhibits higher and fluctuating values (~275 Å^2). (D) Two-dimensional Gibbs free-energy landscapes (ΔG) as a function of RMSD and Rg for Amuc_0517 in complex with pNPGal (left) and pNPGlu (right). The galactoside-bound enzyme populates a single, narrow basin (ΔG = 0 kJ mol^{-1}; RMSD ≈ 0.1 nm, Rg ≈ 2.56 nm), whereas the glucoside-bound enzyme exhibits multiple dispersed basins with higher energy barriers (ΔG ≥ 10 kJ mol^{-1}) and increased Rg (≥ 2.64 nm), indicating greater conformational heterogeneity.
